# Developing and Evaluating Digital Interventions to Promote Behavior Change in Health and Health Care: Recommendations Resulting From an International Workshop

**DOI:** 10.2196/jmir.7126

**Published:** 2017-06-29

**Authors:** Susan Michie, Lucy Yardley, Robert West, Kevin Patrick, Felix Greaves

**Affiliations:** ^1^ Centre for Behaviour Change Department of Clinical, Educational and Health Psychology University College London London United Kingdom; ^2^ Department of Psychology University of Southampton Southampton United Kingdom; ^3^ Nuffield Department of Primary Care Health Sciences University of Oxford Oxford United Kingdom; ^4^ Health Behaviour Research Centre University College London United Kingdom; ^5^ Department of Family Medicine and Public Health The Qualcomm Institute - Calit2 University of California San Diego, CA United States; ^6^ The Center for Wireless and Population Health Systems The Qualcomm Institute - Calit2 University of California San Diego, CA United States; ^7^ Public Health England Skipton House London United Kingdom; ^8^ Department of Primary Care and Public Health Imperial College London London United Kingdom

**Keywords:** health behavior, psychological theory, mobile applications, behavioral medicine, mHealth, eHealth

## Abstract

Devices and programs using digital technology to foster or support behavior change (digital interventions) are increasingly ubiquitous, being adopted for use in patient diagnosis and treatment, self-management of chronic diseases, and in primary prevention. They have been heralded as potentially revolutionizing the ways in which individuals can monitor and improve their health behaviors and health care by improving outcomes, reducing costs, and improving the patient experience. However, we are still mainly in the age of promise rather than delivery. Developing and evaluating these digital interventions presents new challenges and new versions of old challenges that require use of improved and perhaps entirely new methods for research and evaluation. This article discusses these challenges and provides recommendations aimed at accelerating the rate of progress in digital behavior intervention research and practice. Areas addressed include intervention development in a rapidly changing technological landscape, promoting user engagement, advancing the underpinning science and theory, evaluating effectiveness and cost-effectiveness, and addressing issues of regulatory, ethical, and information governance. This article is the result of a two-day international workshop on how to create, evaluate, and implement effective digital interventions in relation to health behaviors. It was held in London in September 2015 and was supported by the United Kingdom’s Medical Research Council (MRC), the National Institute for Health Research (NIHR), the Methodology Research Programme (PI Susan Michie), and the Robert Wood Johnson Foundation of the United States (PI Kevin Patrick). Important recommendations to manage the rapid pace of change include considering using emerging techniques from data science, machine learning, and Bayesian approaches and learning from other disciplines including computer science and engineering. With regard to assessing and promoting engagement, a key conclusion was that sustained engagement is not always required and that for each intervention it is useful to establish what constitutes “effective engagement,” that is, sufficient engagement to achieve the intended outcomes. The potential of digital interventions for testing and advancing theories of behavior change by generating ecologically valid, real-time objective data was recognized. Evaluations should include all phases of the development cycle, designed for generalizability, and consider new experimental designs to make the best use of rich data streams. Future health economics analyses need to recognize and model the complex and potentially far-reaching costs and benefits of digital interventions. In terms of governance, developers of digital behavior interventions should comply with existing regulatory frameworks, but with consideration for emerging standards around information governance, ethics, and interoperability.

## Introduction

Programs and devices using digital technology (digital interventions) have great potential to improve population health and the efficiency and reach of health care delivery. Mobile apps, SMS (short message service) messages, wearable and ambient sensors, social media, and interactive websites can improve health by supporting behaviors involved in disease prevention, self-management of long-term conditions, and delivery of evidence-based health care practice. Such interventions also have potential to do harm if they provide inappropriate advice, involve interactions that undermine desired behaviors, inappropriately share data, or are used instead of more effective behavior change interventions.

Many of these digital interventions seek to foster or support behavior change on the part of health care professionals, patients, or the general public. The challenges involved in developing, evaluating, and implementing effective digital behavior change interventions (DBCIs), and preventing the use of counterproductive ones, have only just begun to be delineated, let alone met [1]. Some of the challenges are similar to those faced by other behavior change interventions, but many are unique, including those of pace of development, engagement with the intervention, measurement of effectiveness and cost effectiveness, and compliance with regulatory, ethical, and security requirements. These challenges are set out in more detail in Table 1.

**Table 1:** Challenges for developing and evaluating digital interventions targeting behavior change.

Topics	Challenges
Pace and efficiency	Rapid technological change and iterative development cycles make it necessary to continually update and adapt interventions.
	Existing development and evaluation cycles are slow and unsuited to dynamic systems and rapidly changing contexts.
	Efficient, continuing relationships between academics and intervention developers are needed for implementation, continued development, and evaluation.
Engagement	Engagement with digital interventions is often too limited to support behavior change.
	Engagement is multidimensional and cannot be evaluated simply by DBCI^a^ usage.
	Engagement with DBCIs may be unequal between different groups and at risk of reinforcing disparities or inequalities.
Theory	Often, there is a lack of clarity around the mechanisms through which DBCIs have their effect.
	Methods of characterizing intervention components, mode of delivery, and contexts that characterize their essential features are required but limited.
Evaluation of effectiveness	Controlling the testing environment is made problematic by the ready availability of alternative interventions.
	It is difficult to specify comparator interventions or control conditions that allow meaningful evaluation of the intervention of interest.
	Better methods for structuring and analyzing very large, dynamic, and heterogeneous data sets are needed.
	Reach and engagement can be low.
	The complex multi-component nature of interventions requires an iterative design and testing cycle.
Evaluation of cost-effectiveness	There is a lack of techniques for economic and cost-effectiveness evaluation across the digital development, deployment, and delivery cycle.
	Funding mechanisms are not aligned with the digital model of development, implementation, iterative improvement, and evaluation.
Regulation, ethics, and information governance	There are competing commercial and ethical demands on data ownership and intellectual property.
	There are emerging and different standards around ethical or institutional review in the biomedical, psychological, and digital development communities.
	There are uncertain quality standards and regulatory processes for digital interventions (with standards either in development or inappropriately adapted from other contexts).

^a^DBCI: Digital behavior change interventions.

There are also unique opportunities. For example, the type and amount of data that can be collected creates unprecedented potential to test and advance theories; understanding more about human behavior will enable the development of more effective DBCIs [2]. Rising to these challenges and making the most of opportunities will require the expertise and collaboration of a wide range of academic disciplines such as behavioral, computer, and engineering sciences and user-centered design.

Given the explosion of development and use of DBCIs aimed at improving health, there is a need for recommendations for designing, evaluating, and implementing digital interventions in health care. Such recommendations are needed to (1) identify the scientific principles relevant to developing effective DBCIs, making digital research more efficient and future interventions safer and more effective and (2) to support key disciplines, health care professionals, patients, and the public to work together more effectively to advance research methods and the understanding and techniques of behavior change through digital technology.

To this end, an international workshop of experts in relevant fields was convened to consider the challenges, opportunities, and strategies for advancement and to formulate principles for developing and evaluating DBCIs. The workshop led to a series of publications [3-7]. This paper discusses key recommendations arising from the workshop and subsequent discussion.

## Methods

An international expert consensus-building two-day workshop, supported by the United Kingdom’s Medical Research Council and the National Institutes of Health and the Robert Wood Johnson Foundation of the United States was held in September 2015 in London. The 42 participants from four countries were selected to include those who develop, evaluate, use, and fund DBCIs for both research and practical purposes. Participants included health care professionals, population health researchers (eg, systematic reviewers, behavioral scientists, and health economists), and intervention developers. The workshop proposal (led by SM) and steering group are shown in Multimedia Appendix 1.

The primary deliverable from this workshop was a set of journal articles that would summarize key issues for research in DBCIs and a synoptic paper setting out some key recommendations. Six topics were identified through discussion amongst the participants. Leaders for each topic were identified and participants were asked to sign up to one or more topics on the basis that they would actively contribute to writing the papers. The topics were (1) the pace of technological development, (2) understanding and promoting effective engagement with users, (3) advancing models and theories, (4) evaluation strategies, (5) economic evaluation, and (6) regulation and governance.

The writing groups met by teleconference before the workshop to write draft papers that were circulated to the whole group in advance of the workshop. These drafts provided the structure and basis of the discussions at the workshop. A formal consensus process was not used, but the structured and open discussions did not reveal any fundamental disagreements about the nature of the recommendations, while at the same time supporting their refinement and specificity. Scribes were appointed from the writing groups to take and circulate notes of relevant points for each paper and the proceedings were audio-recorded. The writing groups developed five articles after the workshop, now published in the American Journal of Preventive Medicine (AJPM) [1,3-7].

The five articles informed the recommendations presented in this article. All authors were workshop participants (SM was PI for the project and LY and KP were Editors of the *AJPM* Special Section).

## Results

The key recommendations for developing and evaluating digital interventions are summarized in Table 2.

### Achieving Rapid and Efficient Development

Behavior change interventions are moving rapidly from their historical roots in interpersonal counseling and mass communication to the increasingly pervasive world of ubiquitous personal mobile and social technologies. Thus, the methods by which we deploy, evaluate, and improve these DBCIs are moving from relatively data-poor, infrequent, and typically post-hoc assessments to methods that incorporate continuous measurement of the intervention effects in real time [3]. Related to these issues, the following recommendations emerged from the discussion.

#### Consider Adopting Methods From Engineering and Other Data-Intensive Domains in the Development Cycle

Rather than using deployment-evaluation cycles in which successive measures are collected every few weeks or months, a new type of rapid feedback approach is possible. Theories, models, and methods to support this approach can be found in systems engineering and related sectors, for example, control theory [8], use of factorial or fractionated evaluation designs [9], and system optimization strategies [10] (please refer to [2] and Multimedia Appendix 2 for more details).

#### Use Bayesian and Related Approaches to Improve the Predictive Modeling Capabilities of Digital Behavior Change Interventions

In addition to enabling more agile evaluation of interventions as they are deployed, sciences outside the traditional medical, public health, and social or behavioral arenas can inform modeling and prediction when multiple behaviors are addressed, when these behaviors have consequences on other important health-related parameters, and for all of these over time and across populations. As with the previous recommendation, the change in scale of the data now attainable from new technologies is analogous to the changes that happened in the field of meteorology when multi-scale and multilevel sensors, combined with improved computing capabilities, enabled weather prediction models to be rapidly built, tested, improved, and retested [11].

**Table 2:** Summary of recommendations according to topic.

Achieving rapid and efficient development	Understanding and promoting engagement	Advancing models and theories	Evaluating effectiveness	Evaluating cost-effectiveness	Ensuring regulatory, ethical, and information governance
Consider adopting methods from engineering and other data-intensive domains in the development cycle.	Specify and establish empirically what constitutes “effective engagement” for each DBCI^a^, that is, sufficient engagement to achieve the intended outcomes.	Use the large amounts of real-time, ecologically valid data generated by DBCIs to test and advance models and theories of behavior change.	Evaluate at all phases in the development cycle.	At every stage, including concept development, identify all the relevant future costs and benefits.	Ensure compliance with appropriate ethics or institutional review board processes.
Use Bayesian and related approaches to improve the predictive modeling capabilities of DBCIs.	Identify and develop valid and efficient combinations of objective and subjective measures to build and test multidimensional models of engagement.	Develop methods able to efficiently analyze large, complex data sets to test dynamic theoretical propositions and allow personalization of DBCIs.	Design evaluations for generalizability.	Take account of projected uptake as well as reach.	Identify and adhere to regulatory processes that may be required for digital medical devices.
Leverage advances in data science such as machine learning, but ensure that human input is retained as needed.	Develop DBCIs with a person-centered and iterative approach, using mixed methods to progressively refine the DBCI to meet user requirements.	Specify the circumstances in which a proposed mechanism of action of a DBCI will produce a targeted effect and build an ontology to organize knowledge resulting from this.	Use methods of DBCI evaluation that capitalize on their unique characteristics.	Select a modeling framework appropriate for the complexity of the projections.	Ensure compliance with national standards for data handling, sharing, and interoperability, where appropriate.
		Develop DBCIs using a modular approach.	Use features of DBCIs to optimize control and access rich data streams.	Separately evaluate societal, personal, and health care cost-effectiveness.	Provide clear and transparent information on how data from the intervention will be used and shared.
		Support interdisciplinary research collaborations and transdisciplinary thinking.	Choose comparators that minimize contamination.		

^a^DBCI: Digital behavior change interventions.

#### Leverage Advances in Data Science Such As Machine Learning, But Ensure That Human Input Is Retained As Needed

Machine learning and related approaches are increasingly being used to solve big data challenges, including health behavior assessment and interventions [12]. This is particularly the case in the move beyond “on average” effects to personalized inputs and outputs based upon each individual’s situation, characteristics, and desired outcomes. However, we are in the early phase of this new science, so the optimal balance between computer-driven processes and human input is not yet clear: a mix may be needed with the balance determined by both qualitative and quantitative assessments of outcomes.

### Understanding and Promoting Engagement

The novel ways in which interventions can be delivered using digital technology result in new ways of engaging with them. Face-to-face behavior change support typically requires users to attend a set number of therapeutic or coaching sessions, whereas users of DBCIs can access support when they feel it is necessary. Analysis of this very different pattern of engagement requires careful consideration of the relationship between the “micro” level of immediate engagement with the digital dimension of the intervention and the “macro” level of engagement with longer-term behavior change (see next section) [13].

#### Specify and Empirically Establish What Constitutes “Effective Engagement” for Each Digital Behavior Change Intervention, That Is, Sufficient Engagement to Achieve the Intended Outcomes

Acknowledgement of the complex relationship between engagement with the behavioral and technological aspects of the intervention challenges the common assumption that engagement can be measured simply by technology usage. Behavior change may or may not require sustained or in-depth engagement with the digital intervention; hence, technology usage correlates with behavioral outcomes. However, the association is often not strong. For some users and contexts, just one in-depth period of engagement with the DBCI may be sufficient to initiate new habits or teach new skills, whereas for other types of behavior change or other users, brief but timely context-triggered prompts may be needed long-term, whenever the behavior is required [14]. It is therefore important to empirically establish what the “effective” engagement required to achieve behavior change is, in a particular intervention context and for any particular user, as this is likely to differ for different types of interventions and target behaviors.

#### Identify and Develop Valid and Efficient Combinations of Objective and Subjective Measures to Build and Test Multidimensional Models of Engagement

Measuring effective engagement requires a multi-dimensional, mixed method approach, combining objective assessment of technology usage, behavior, and reactions to the intervention with reports of subjective and offline experiences of users. DBCIs promise exciting new opportunities to collect detailed objective longitudinal data about the antecedents of behavior change, but much work is necessary to develop and validate reliable, non-intrusive means of assessing and analyzing user behavior and its context. Qualitative methods are resource intensive but provide vital complementary insights into user views and behaviors [15]—for example, when not engaging with the digital dimension of the intervention.

#### Develop Digital Behavior Change Interventions With a Person-Centered and Iterative Approach, Using Mixed Methods to Progressively Refine Them to Meet User Requirements

To promote engagement with DBCIs, a “user-centered” [16] or “person-based” [17] approach is essential to ensure that interventions are responsive to users’ needs and preferences. These approaches are useful in the development of any intervention, but are particularly important for developing DBCIs; human therapists can adjust their advice in real time based on user reactions, whereas the content and delivery of DBCIs must be pre-adapted during development to anticipate a range of user reactions. This involves carrying out iterative qualitative research and stakeholder consultation throughout the design and development process. Tailoring interventions to different needs and preferences can improve engagement, but a well-designed intervention that allows choice is often accessible and engaging for a wide range of users. Adding human support is also known to promote engagement with many interventions [18]. However, as tailoring and human support both increase the cost of interventions, it is important to establish when and how interventions need to be tailored to the individual or supplemented by human support.

### Advancing Models and Theories of Behavior Change

Digital technology makes it much easier to collect data in real time and places less reliance on self-report when it comes to recording behavior and taking physiological or physical measurements of study participants or their environment. DBCIs generate large amounts of real-time, ecologically valid data that form digital traces that can be aggregated, connected, and organized to gain greater understanding of how and why behavior changes within an individual over time and how that is influenced by internal physiological and psychological states and the external world. To realize the potential of these data for understanding and changing behavior, a number of recommendations emerged from the discussion (see [4]).

#### Use the Large Amounts of Real-Time, Ecologically Valid Data Generated by Digital Behavior Change Interventions to Test and Advance Models and Theories of Behavior Change

Data should be collected at a level of granularity that enables the testing and advancement of models and theories of behavior change, accounting for individual variation and changes over time. These data should be used to build dynamic theories of human behavior, modeling not just causal and mediating relationships, but accounting for how effects vary across individuals, contexts, and over time. Theories and models should be continually tested by DBCI-generated data and the results used to systematically refine models and theories.

#### Develop Methods to Efficiently Analyze Large, Complex Data Sets to Test Dynamic Theoretical Propositions and Allow Personalization of Digital Behavior Change Interventions

Achieving the promise of DBCIs for advancing behavior change theories requires methods that allow vast amounts of complex data to be analyzed and interpreted. For theories and models to be useful in guiding data analysis and interpreting findings, they should be as precise, quantitative, and testable as possible [19]. This allows the building of idiographic models of behavior change and the personalization of DBCIs, that is, the tailoring of the DBCI content and delivery to individuals. It allows DBCIs to adapt, as data are gathered about how the person responds in different contexts. It also enables DBCIs to intervene at opportune moments (sometimes referred to as ecological momentary interventions [20] or “just-in-time” adaptive interventions [14,21].

#### Specify the Circumstances in Which a Digital Behavior Change Intervention’s Proposed Mechanism of Action Will Produce a Targeted Effect and Build an Ontology to Organize Knowledge Resulting From This

DBCIs represent a qualitative leap in our ability to answer the question posed by researchers, practitioners, and policy-makers: “What works for whom in what settings to change what behaviors, and how?” To optimally organize knowledge about the circumstances (when, where, for whom, and in what state for that person) in which a proposed mechanism of action for a DBCI will produce a targeted effect requires coherent structures and uniform terminologies to describe constructs and their inter-relationships [2,22]. Such knowledge-organizing structures are called “ontologies” [22,23]. Work is beginning to develop an ontology of behavior change interventions. The Human Behaviour Change Project is applying artificial intelligence to refine an ontology for analyzing the up-to-date world literature to answer the question “What works, how well, with what degree of exposure, for whom, in what settings, with what behaviors, and why?” [24].

#### Develop Digital Behavior Change Interventions Using a Modular Approach

To optimize the effectiveness of DBCIs, theory should be applied to their development and the data generated should be analyzed in terms of the underlying theoretical propositions in order to test and advance theory. This can be done by a modular approach to DBCI development in which modules represent one or more specific theoretical propositions [14]. An example of this approach is “Drink Less,” a mobile app [25] to reduce excessive alcohol consumption in which five theoretically distinct modules were tested in a full factorial trial (Normative feedback, Feedback and self-monitoring, Identity change, Action planning, and Cognitive bias re-training) [26]. This allows the linking of theory to intervention content, the investigation of which techniques and mechanisms is doing the “heavy lifting” in effective interventions and enables the cumulative process of advancing theory and developing more effective DBCIs. Testing and advancing theory is strengthened by supplementing these quantitative data analytic strategies with qualitative research methods.

#### Support Interdisciplinary Research Collaborations and Transdisciplinary Thinking

Ontologies help both to organize evidence and strengthen interdisciplinary collaboration in developing and evaluating DBCIs informed by models and theories of behavior change. Such collaborations, for example between behavioral scientists, engineers, and computer scientists, will bring unique expertise to bear upon the challenges described in this paper, but also, in the right circumstances, generate new understandings and knowledge that cross disciplinary boundaries, with the whole greater than the sum of the parts [27].

### Evaluating Effectiveness

Evaluating effectiveness can be challenging for any behavior change intervention because of problems obtaining valid outcome measures and sufficient numbers of participants who are representative of the population of interest, fidelity in terms of delivery of the intervention, varying levels of engagement with the intervention, loss to follow up, and context sensitivity of the findings. Evaluating DBCIs presents a particular blend of challenges, some similar to other behavior change intervention modalities and some different. Here, we highlight some key recommendations (see [5]).

#### Evaluate at all Phases in the Development Cycle

Evaluation must be built into the development cycle from the initial concept, and then carried through to prototypes and the final implemented version. This involves concept testing, user testing, factorial experiments, randomized controlled trials (where feasible and appropriate), and testing against baseline after implementation (A-B testing) [2]. For an extensive list with definitions of options for DBCI evaluation, please see Multimedia Appendix 2, drawn from a published guide to developing and evaluating DBCIs in health care [2].

#### Design Evaluations for Generalizability

Given the high context sensitivity of DBCI effectiveness, evaluations need to be set up in such a way that inference beyond the strict testing conditions can be justified. Generalization may be to different types of populations, the same type of population at a later date, or a different implementation of the DBCI. For example, rapidly changing fashions with regard to design and use of interactive components means that studies need to be designed to allow plausible generalizations beyond specific DBCI features. Qualitative research has a contribution to make in identifying and informing aspects of delivery and context that increase understanding of generalizability.

#### Use Agile Methods of Digital Behavior Change Intervention Evaluation That Capitalize on Unique Characteristics

The continuous and data-intensive nature of DBCIs, combined with the rapidly changing technologies that support them, challenge traditional research designs such as randomized controlled trials (RCTs) with locked-down interventions and research approaches that involve lengthy recruitment, enrolment, and study periods. While RCTs will continue to have value, they should be complemented with adaptive research designs, A/B testing, N-of-1 studies, and other research methods that yield insights in a shorter time frame or in ways that reflect the granular nature of the intervention effects [28]. DBCIs also provide the opportunity to engage the user directly in the design of the intervention, including tailoring of preferences based upon context and changing life circumstances.

#### Use Features of Digital Behavior Change Interventions to Optimize Control and Access Rich Data Streams

DBCIs can deliver complex personalized interventions with high fidelity, and engagement with their components can be assessed automatically. This provides an unrivalled opportunity to undertake factorial experiments to assess the effectiveness of components, but presents major challenges in terms of conceptualizing and analyzing very large temporally structured data streams. Analytical methods need to be devised to address this challenge, but their value will be limited without a theoretical underpinning to structuring and aggregating data.

#### Choose Comparators That Minimize Contamination

For many DBCIs, such as smoking cessation or weight management apps, highly developed products are readily available to study participants. Therefore, researchers face a difficult choice between a comparator that has sufficient active components to be credible and deter searching for alternatives and one that has so much active content that the “true” effect of the intervention cannot be measured. In practice, for many DBCIs, we have to accept that we may never be able to assess their full impact, only their impact relative to another active intervention.

### Evaluating Cost-Effectiveness

Rigorous economic evaluation is required to inform decision makers about allocation of their scarce resources. A central motivation for DBCIs is that they may be cost effective—and certainly cheaper than their face-to-face counterparts—because they can rapidly increase scale at minimal cost. Generating accurate evidence of cost effectiveness will be essential in gaining support from health system payers, but it requires additional consideration beyond conventional analysis (see [7]).

#### At Every Stage, Including Concept Development, Identify all the Relevant Future Costs

As with all interventions, economic evaluation should be considered from the start of intervention development to ensure that all cost data are collected. However, existing economic approaches (eg, ISPOR guidance [29]) will need to be adapted to take into account the way digital health interventions are delivered, covering the whole life cycle of the intervention. This may include development, implementation, update, and eventual obsolescence. While scaling interventions may be cheap, with a low additional unit cost, development costs can be high at the start of the process and need to be fully included in calculations. The rapid cycle of development for many DBCIs, with multiple versions of a product that evolves with small iterative and incremental improvements, is different from that of the relatively fixed products common in the pharmaceutical and medical devices industries. The cost of these iterations must be captured and included across the lifespan of the intervention. In addition, as many DBCIs may be more effective when supplemented with human interactions, evaluations may need to consider these less cheaply scaled human costs.

#### Take Projected Uptake and Reach into Account

Economic evaluations will need to understand issues of uptake, reach, and retention of the intervention, as these will be central factors in any projection of benefit. Consideration will also need to be given to the effects of interventions around those immediately engaged, including effects on wider social networks. For example, given the typically high development costs of a DBCI, the cost-effectiveness can depend critically on getting a large number of users. Economic evaluations need to be able to draw on evidence as to the likely uptake of a DBCI to assess this, which may include costs incurred in promoting the DBCI to help drive uptake.

#### Select a Modeling Framework Appropriate for the Complexity of the Projections

DBCIs are complex interventions by nature; they are responsive, with multiple components. Economic appraisals will need to recognize this complexity—in terms of interventions, outcomes, and causal pathways [30]—and use appropriate methods [31] to assess them. More complex interventions may require the use of sophisticated modeling techniques, including agent-based approaches, which capture dynamic interactions between the intervention, the population it is applied to, and the wider environment.

#### Separately Evaluate Societal, Personal, and Health Care Cost-Effectiveness

Finally, cost effectiveness analysis must be diligent in measuring effect across its forms. This might include benefit (1) to an individual, for example, in improved quality of life, (2) to society, for example, in improved productivity of the workforce, and (3) to the health care system, for example, in reduced referrals to conventional face-to-face settings or hospital admissions. Ideally, disaggregation of these separate components should be attempted, if possible.

### Ensuring Regulatory, Ethical, and Information Governance

Beyond the practical and measurable aspects of development and evaluation, DBCIs present new questions in terms of ethics and regulation as they challenge existing frameworks and rules often designed in a pre-digital era.

#### Ensure Compliance With Rules on Ethics or Institutional Review Board Processes

The diversity of disciplines involved in the development of new DBCIs is challenging as the expectations and approaches of different cultures involved may not be immediately aligned. The notion of a minimum viable product and “fail fast, fail often” might be encouraged in an engineering world view, but it stands in sharp contrast to the “first do no harm” spirit of the health care profession and the risk-averse nature of clinical governance and patient safety. Developers may need to adjust to the more stringent regulatory frameworks of the biomedical sector and local ethics or institutional review processes. At the same time, regulators will need to make improvements in terms of speed and responsiveness in order to be able to meet the needs of fast evolving technologies. This was emphasized in the United States in a 2016 report from the National Academies of Science, Engineering, and Medicine [32], which advocated reflecting the contemporary realities of new research contexts, including accessibility and use of personal data.

#### Identify and Adhere to Regulatory Processes That May Be Required for Digital Medical Devices

DBCIs will need to navigate the complex landscape of regulation and governance. Many of these interventions fall at the blurred edges of existing regulatory frameworks, which evolved in a pre-digital and less software-intensive era. Depending on their nature, many DBCIs will be classified as medical devices. A lack of consistency of approach between countries means that those developing interventions will need to carefully monitor and engage with the emerging frameworks of the jurisdictions they work within. Regulatory organizations include the United Kingdom’s Medicines & Healthcare Products Regulatory Agency, which has issued specific guidance on apps [33] and the US Federal Trade Commission, which has released similar advice [34]. In addition, although several frameworks for assessment exist, there is diversity in views about what the necessary domains of quality are for DBCIs. Developers will need to understand this variation and create the appropriate evidence to match these specifications. Developing assessment frameworks in the United Kingdom include the National Health Service (NHS) app assessment model [35] and the European Union mHealth assessment guidelines [36].

#### Ensure Compliance With National Standards for Data Handling, Sharing, and Interoperability, Where Appropriate

There are unique challenges of information governance, as DBCIs may produce large amounts of personal, identifiable, and potentially valuable data. As data is often collected directly from mobile devices, this may include geographical information or information related to contacts and social networks. Those developing interventions will have to comply with local legislation that varies in scope and detail. Relevant legislation includes the Health Insurance Portability and Accountability Act (HIPAA) [37] in the United States, the Data Protection Act [38] (together with the Caldecott principles) in the United Kingdom, and the General Data Privacy Regulation in the EU [39]. In addition, DBCIs may have more benefit if they are able to formally interact with health care delivery systems and, in particular, with electronic health records, requiring compatibility with local (and often highly variable) standards.

#### Provide Clear and Transparent Information on How Data From the Intervention Will Be Used and Shared

Beyond the essential regulatory frameworks, there are wider societal questions that have not yet been definitively answered regarding acceptability of data collection and sharing with competing commercial and ethical demands on data ownership. Some interventions are provided on a fee-for-use basis. Others have more complicated business models where a product is provided for free, but data is made available to the provider. There may be a trade-off that the public is willing to make between sharing their own data, effectiveness of interventions, and cost. As the threshold of acceptability is still being understood—and as this threshold may vary between populations and individuals—developers will need to tread cautiously and transparently. In addition, ownership of the data produced by DBCIs is not always clear and may vary by jurisdiction. These data have the potential to be a valuable resource for health care research and service planning [40], but the public may not be aware of these potential uses and may have reservations about how the data are used [41]. Developers should provide clarity about how and with whom they share their data. They may also wish to make sure that the business model for provision of their intervention is openly reported.

## Discussion

### Future Research

The approaches used to address the compelling health problems of our times should be based in state-of-the-art science in medicine, public health, and the social and behavioral sciences. They must also reflect advances in other disciplines such as engineering, data science, systems science, human-computer interaction, and communications technologies, in particular if these promise to improve the means by which we both understand the determinants of health and improve health behaviors. Innovations in digital health are now emerging on several fronts that demonstrate the utility of methods drawn from these fields. This should not surprise anyone who is engaged as a digital citizen in the increasingly smart and connected world in which we now live. The mobile in our pockets, purses, or backpacks is becoming both an increasingly important window on the potential of digital health and an enabler thereof. Thus, there is a need to consider several implications of these developments and optimally shape them as they go forward.

The ways in which users engage with digital interventions are changing as fast as the technologies for delivering them, and it is important that our methods of conceptualizing and assessing engagement keep pace. Earlier, PC-based digital interventions often replicated a series of therapeutic or coaching “sessions,” and engagement was defined simply as completing sufficient sessions to receive the full therapeutic program. However, mobile users of just-in-time adaptive interventions may just engage briefly at the crucial time-point with precisely the required behavior change support. The “effective” engagement required for behavior change support is therefore likely to differ, depending on the users and their contexts, and can only be determined by analyzing complex patterns of relationships between usage, user experiences, and outcomes. A challenge for future research into DBCIs is to find the most valid and efficient combinations of methods of measuring engagement with the intervention, both on the Internet and offline. Technology can provide detailed, unobtrusive assessment of behavior and its context, while complementary qualitative methods are crucial to fully understand and interpret user experiences. These qualitative approaches are central to participatory user-centered design, which is the key to developing and evaluating DBCIs in order to ensure that they are engaging and effective [16,17].

With respect to theory, DBCIs provide an unprecedented opportunity to test and advance our understanding of how behavior changes. However, this requires the application of more “agile” science [42]. This includes designing DBCIs on a modular basis with a clear understanding of which behavior change techniques [43] constitute that module and how they link to the theoretically postulated mechanisms of action. Collaborations between behavioral scientists and computer scientists are enabling theories of change to be specified in precise detail as sets of constructs and specified relationships between them. Computational models can define and test not just interactions between modules, mechanisms of action, and behaviors, but also how these vary across individuals and populations, settings, and time. This is spawning a fruitful area of “ontology” development, ontologies being well specified structures for organizing knowledge [22]. By combining this with computational algorithms and machine learning, the promise is that we can advance theoretical understanding of behavior change so that we can answer with confidence the questions put by health practitioners and policy makers: “What works, how well, for whom, in what settings, for what behaviors, and why?” [24].

With respect to evaluation, one key effectiveness question for policy makers and practitioners is whether purchasing or commissioning a given intervention will improve outcomes for their population compared with current practice or another innovation that they may be considering. Another is whether digital interventions can reduce the cost of health care provision without a significant loss of effectiveness. Ideally, this would involve pragmatic randomized trials (individual or cluster) comparing the different forms of care. However, the analysis in this paper indicates that this is often impracticable. Moreover, there are so many unknowns in the development of these interventions that a much more iterative cycle of development and testing is needed before getting to the point of a pivotal evaluation. Even when an intervention has been found to be effective in a pivotal evaluation, there are no guarantees that it will continue to be effective in a rapidly changing context. This means that the evaluation strategy for DBCIs needs to be fully integrated into every phase of development and implementation and needs to take advantage of the wide range of methodologies that are available, both experimental and observational, while also taking into account the exposure as well as effect in those exposed. The evaluation needs to take advantage of statistical techniques, particularly Bayesian analyses that promote ongoing accumulation of evidence.

With respect to the economic evaluation, policy makers and service providers will be hungry for the potential to deliver interventions at scale with only small increases in marginal cost, often in comparison with face-to-face interventions. This creates a requirement for evaluators to adapt existing economic approaches and develop new ones to accurately capture cost across the full digital development cycle, measure the reach of the intervention, and consider the complexity of the intervention to allow meaningful comparison. In evaluating both effectiveness and cost effectiveness, policy makers and organizations that consider purchasing these services will need to compare them with conventional delivery models and evaluation approaches. Thus, developers and evaluators will need to ensure that they produce evidence that allows this comparison to be made.

With respect to ethical, regulatory, and policy issues, standards for DBCIs are still emerging, and people developing and testing them will have to pick a cautious path through rules not often designed with this new category of interventions in mind. They will need to ensure that new, agile approaches to testing DBCIs demonstrate that they meet the exacting expectations of the clinical and biomedical community. Possibilities to develop common standards for intra-operability between interventions, with electronic health records and health care delivery systems, and in common behavioral data repositories should be explored.

The advice provided here is specific to DBCIs, but it should also build on the good practice of reporting general mHealth interventions published elsewhere to ensure adequate description of the technical components of the intervention [44,45].

### Conclusions

DBCIs present unique methodological challenges. New techniques and approaches are becoming available that offer opportunities to accelerate both the development and evaluation of these interventions, taking advantage of the speed and volume of data they generate, their potential adaptability, reach, and cost. These new methods have implications for changes within other fields of health care.

## Appendix

Multimedia Appendix 1 How to create, evaluate, and implement effective digital healthcare interventions: development of guidance.

Multimedia Appendix 2 Options for testing DBCIs.

## Abbreviations

DBCI: digital behavior change intervention
EU: European Union
HIPAA: Health Insurance Portability and Accountability Act
MRC: Medical Research Council
NHS: National Health Service
PI: Principal investigator
RCT: randomized controlled trial
