# Supplementary material for: Developing and Evaluating Digital Interventions to Promote Behavior Change in Health and Health Care: Recommendations Resulting From an International Workshop
Source: J Med Internet Res. 2017 Jun 29;19(6):e232. doi: 10.2196/jmir.7126 (PMC5509948; doi:10.2196/jmir.7126)
Supplement: Multimedia Appendix 1 [file jmir_v19i6e232_app1.pdf]

Multimedia appendix 1

How to create, evaluate and implement effective digital healthcare interventions: development of guidance.

International workshop, London UK, 10-11 September 2015
Leading to published guidance and accompanying articles/editorials

Sponsored by
- Medical Research Council (MRC), UK
- The National Institutes of Health (NIH) Office for Behavioral and Social Sciences Research (OBSSR), USA
- The Robert Wood Johnson Foundation, USA.

Proposers
Susan Michie, Professor of Health Psychology and Director of the Centre for Behaviour Change, University College London, UK & Jeremy Wyatt, Professor of eHealth research, University of Leeds, UK.

Introduction

Digital interventions that use smartphone Apps, SMS messages, sensors and websites have huge potential to improve population health and the efficiency of healthcare delivery and engagement. This can be achieved by supporting behaviours involved in disease prevention, self-management of long-term conditions and delivery of evidence-based healthcare practice. They also have potential to do harm if they provide inappropriate advice, involve interactions that undermine desired behaviours or are used instead of more effective behaviour change interventions.

The challenges involves in developing, evaluating and implementing effective digital behaviour change interventions, and preventing use of counterproductive ones, have only just begun to be delineated, let alone met. Some of the challenges are similar to ones faced by other behaviour change interventions, but many are unique or at least uniquely acute.

Among these challenges are: rapid technological change making interventions obsolete before they can be adequately evaluated; impossibility of controlling the testing environment because of ready availability of alternative interventions; specifying comparator interventions or control conditions that allow meaningful evaluation of the intervention of interest; promoting effective engagement with digital interventions; finding effective synergies between digital and person-delivered interventions; establishing efficient, continuing relationships between academics and developers needed for implementation and continued development; collaborative development of models and theories required by these interventions; developing methods of characterising intervention components, mode of delivery and context that characterise their essential features; establishing appropriate funding mechanisms to support implementation and continued development; the need for methods for structuring and analysing very large,

dynamic data sets; establishing intellectual property in the context of competing commercial and ethical demands; development of quality standards for digital interventions.

There is thus a pressing need for new guidance for designing, evaluating and implementing digital interventions in healthcare, drawing on the expertise from a range of academic disciplines such as behavioural, computer and engineering sciences and from the user angle. We believe that new, cross-disciplinary and consensus-based guidance is needed to (i) identify the scientific principles of developing effective digital interventions, making digital research more efficient and future interventions safer and more effective, and (ii) support key disciplines to work together more effectively to advance research methods and the understanding and techniques of behaviour change. The idea for a workshop to bring together leaders in the field to consider this problem and to formulate guidance on the principles for developing and evaluating such interventions was initiated within the UK Medical Research Council's Methodology Panel and has subsequently attracted wider support both in the UK and internationally.

Definition & scope

Digital interventions are tools to help the population reduce health-related risks, help them manage long -term conditions or improve their access to healthcare, and to enable health professionals to be more effective and deliver evidence-based practice (e.g. smartphone apps or internet interventions to be used personally or in collaboration with patients). These interventions are known by a variety of terms, including eHealth, mHealth, mobile technology, computer-based, and include: social media, mobile apps, wearable and deployable sensors, internet/web sites (eg. online forums, risk calculators, charting tools), SMS programmes for health promotion, telehealth, serious games and decision support systems. These technologies can capture contextualised health-related behaviour in real-time (e.g., location, environment, social situation, mood), and have the potential to mesh a broad variety of personal and public data to understand behaviour. They can map responses to intervention type and dose in a near continuous fashion, providing the opportunity to adapt and personalise interventions on the fly. They therefore offer unprecedented avenues to understand and influence behaviour 'for good'. The scope focuses on interventions to change individual behaviour rather than on organisational systems such as electronic patient records or ePrescribing systems.

Methodological challenges presented by digital health interventions

Of the challenges identified above, six appear to have a specially high priority in terms of methodological guidance: the rapid rate of technological development, defining the nature of the intervention and comparator conditions, the potential to test theory collaboratively, the problems of engagement with new technology, how best to combine new technology with human input, and how best to generate quality standards.

Developing methods that are 'fit for purpose' given these challenges will require the coming together of a range of academic disciplines, including behavioural, engineering and computer sciences, and including human-computer interaction and user perspectives. There is not a rich tradition of close working across these disciplines and an urgent need to foster cross-disciplinary understanding, thinking and collaboration.

This will include understanding each other's concepts and language, and generating consensus definitions so that different terms are not used to mean the same thing or the same term used to mean different things. Below are some key challenges:

1. **Addressing rapid obsolescence**

- Early/ongoing evaluation of interventions needs to allow for a variety of interim forms of evaluation, such as multiple n-of-1 studies, feasibility studies, and implementation studies 'in the wild' – typically using interim and self-report outcomes. In addition, new methods of rapid but robust evaluation need to be developed, for example, rapid cycles of A/B experimental testing on proximal measures that allows the more effective condition to become the new control condition in an on-going process.
- To be efficient, this process needs to be in the context of a relevant evidence-based theory of change
- These interim evaluations need to be co-ordinated with fewer, longer-term sufficiently powered RCTs of behavioural and/or health outcomes and cost-effectiveness. These will be able to evaluate the sustainability of outcomes, and also the validity of the predictive model underpinning the rapid A/B testing.
- Because delivery technology is rapidly changing, there is a need to evaluate underlying principles of the intervention (eg. to uncover enduring principles about how to design persuasive content) that can be taken forward across different forms (technologies or communication channels). Although not usually thought about in the same way, this is similar to the evaluation of more conventional interventions in which the form (e.g. setting, modes of delivery) can vary from trial to translation conditions.
- The rapid change of technology raises the question as to what constitutes a sufficiently important change to warrant re-evaluation and how this is best funded, given ongoing and sometimes unpredictable programming costs.
- In view of the above points, experimental approaches should be accompanied by in-depth iterative qualitative development with users, including but going beyond usability testing.
- To keep up with the pace and applications of technological development, there is a need for cross-disciplinary teams and communication, which requires learning about each others' concepts and terminology.

2. **Identifying appropriate intervention and comparator conditions**

- Standard randomised controlled trial methods derived from pharmaceutical research are predicated on having highly specified intervention and comparator conditions. There has been growing awareness of the need for detailed specification of interventions and comparators in complex intervention research overall, but this learning can be hard to apply to digital interventions.
- The intervention may be hard to define precisely if there is a high degree of tailoring, adaptive learning and user choice. There will be increasingly few interventions that have a 'standardized' intervention protocol in the conventional understanding of 'standardised'. We might have standardised decision nodes, but those will lead to different interventions (content as well as dose) for different people. Our current theories, methodologies and statistical methods are not necessarily up to the task, although research designs such as SMART and MOST can help us here to some extent.
- Similarly, the comparator conditions for digital interventions are more complicated than "treatment as usual" as the participants in the comparator group may have access to a myriad of other digital

interventions. Selection of a suitable comparator requires detailed analysis of the specific research questions in relation to both the effect and the mechanisms of change that are being evaluated.

- The tension between external and internal validity of a trial appears to be particularly acute in evaluation of digital interventions. Actions taken to enhance internal validity, including e.g. participant identity validation, obtaining off-line contact details to promote good follow-up rates, or "run-in" periods prior to trial entry are particularly likely to skew participant populations and jeopardise external validity. This may have significant impact on the generalizability of trial results to the use of such interventions in routine practice.
- There is increasing evidence that completing measures to provide information about mediators and moderators can by itself bring about change (a kind of Hawthorne Effect). Possible solutions are to use digital technology to collect routine data unobtrusively and/or to include a control with no measures and a control with minimal measures and qualitative data to elucidate mechanisms of action.

3. **Collaborative development of appropriate models and theories**

- The multi-component nature of digital interventions along with their large reach and therefore power to detect differences between people and changes within people in real time provides the potential to develop and test theory collaboratively, using sophisticated, theory-informed designs such as fractionated factorial study designs, with selected cells guided by theory and supplemented with usage data analyses and think aloud techniques (Collins et al, 2007).
- Cross-site and cross discipline collaboration to test the effectiveness of and share modules within digital interventions and applications across populations and settings.
- The unprecedented streams of on-going, temporally dense and contextually rich data will allow us not only to (re)develop theories to understand behaviour in time and in context, but to transform these theories into testable computational models . The field needs these new models to be able to guide Just-In-Time, Adaptive Interventions JITAIs) that new technologies can now support (Riley et al Spruijt-Metz and Nilsen 2014).
- Modelling new variables that might emerge from the unprecedented data streams that mHealth provides, which may be extracted by engineers and identified by behavioural and social scientists.
- Integrating understanding and thinking across academic disciplines and between academia and other sectors to provide a step change in theories and methods of behaviour change, and their application to intervention development and evaluation

4. **Optimising engagement**

- Sustained engagement is a major problem with many digital health interventions; often it proves relatively easy to get people to download & use digital interventions for a short period, but difficult to retain them for long enough to make a difference to health.
- Despite the impressive potential reach of digital interventions, often they are taken up mainly by those with better education, health and health literacy. We need to ensure that interventions do not increase the 'digital divide' and health inequalities.
- There is a need to develop the conceptualisation and operationalisation of engagement, including conceiving engagement itself as a behaviour which should be studied in the same way as other behaviours. This means behavioural scientists engaging with human-computer interaction experts and those with expertise in interactive media, design and visual arts.
- One size of user interface (or one design) does not necessarily fit all. mHealth tools may need to be tailored to 'fit' different populations (culturally sensitive, user-centered design) e.g. according to culture, age group, healthcare needs – or opportunities can be provided to allow 'self-tailoring' by

the users. Equally, it is important to identify design features that are useful (or not useful) for most populations, to avoid unnecessary development costs (especially for interventions targeted at populations with less prevalent health conditions and so less potential for economies of scale).

- With increasing pressure on healthcare budgets and the increasing potential of new technologies to support delivery and self-management of healthcare, new models of human-technology interaction need to be investigated. This poses new methodological challenges as to how to conceptualise and operationalize research questions addressing how to achieve the most effective combinations of human and technological input.

5. **How best to undertake health economic analyses of digital interventions.**

- The current literature has very little on the costs and cost-effectiveness of digital interventions. We need to correct this, given that one of the major drivers for digital interventions is their presumed cost-effectiveness.
- Guidance is needed on what costs to include in a health economic analysis (e.g. all the costs of development or just the costs of maintenance and support), as well as the importance of including cost-benefits in evaluations (e.g. reductions in consultation rates, improvements in health outcomes).

6. **Developing quality standards**

- There is considerable concern about the variable quality of digital health interventions and some current dangerous practice (eg. 15 fraudulent alcohol content calculators, including drinkers being told to breathe on their
- mobile phone and being told they are in safe limits). This raises issues of ethics, standards and regulation.
- The consumerist attitude to digital interventions and assumptions around the value of the free market threatens their reputation and is a barrier to high quality development
- A number of approaches could help improve the quality of digital interventions (eg. professional and public reviews, curated app stores, various approaches to regulation, empowering developers to evaluate their apps) but it is important that these do not stifle creativity
- A prototype set of 24 quality criteria with suggested evaluation methods for smartphone apps has been developed by Wyatt and colleagues that would form a starting point for this discussion

Benefits of guidance in this area

The table summarises the potential added value of such guidance, by stakeholder:

**Stakeholder**	**Benefits**
Researchers evaluating the intervention	An authoritative framework that guides the type of study to be carried out during successive evaluation phases<br>A clear language with which to describe and formulate research and evaluation plans and stages<br>Guidance on what kind of expertise should be present in study teams
Research funders	A framework that funding applicants can use to structure their digital intervention proposals, and for referees to assess proposals against<br>Fewer proposals for evaluations of digital interventions that are not yet ready for evaluation

	Fewer negative evaluations that fail to contribute usefully to our theory of digital interventions
Intervention developers	A structured framework that clarifies the development stages for digital interventions<br>Greater chances of success with their intervention
Clinicians using the intervention	An authoritative framework to guide clinician's questioning of system developers about the results of successive evaluation phases
Journal editors and referees	A framework for authors to use to structure their digital intervention articles, and against which referees can assess them
Patients	Reassurance that a higher proportion of digital interventions will bring health benefit
The public, taxpayers & policy makers	Reassurance that most digital interventions used in the public sector bring health benefit
NHS commissioners	A framework for judging proposals by providers to introduce new or expensive digital interventions into routine use

Method

1. **A 1.5/2-day workshop** of up to 40 scientists (behavioural, engineering and computing), methodologists and digital intervention experts to discuss key issues and content of guidance. Consider involving policy makers, funders and those who implement interventions, either within this workshop or as a follow-up
2. **Working groups** to prepare papers for the workshop and draft guidance iteratively, presenting and circulating to workshop participants for feedback.

Steering group:

- Prof Susan Michie (Principal Investigator): s.michie@ucl.ac.uk
- Prof Jeremy Wyatt (co-PI): j.c.wyatt@leeds.ac.uk
- Prof Lucy Yardley, University of Southampton: L.Yardley@soton.ac.uk
- Prof Robert West, UCL Tobacco and Alcohol Research Group: robert.west@ucl.ac.uk
- Prof Elizabeth Murray, Director of eHealth Unit, UCL: elizabeth.murray@ucl.ac.uk
- Prof Mike Kelly, head of Public Health at NICE: mike.kelly@nice.org.uk
- Dr David Crosby (MRC): David.Crosby@headoffice.mrc.ac.uk
- Sean Bamberger (Centre for Behaviour Change to provide administrative support): s.bamberger@ucl.ac.uk
- Dr Bill Riley, National Institutes of Health, USA: wiriley@mail.nih.gov
- Dr Kevin Patrick, University of California at San Diego: kpatrick@eng.ucsd.edu
- Dr Donna Spruijt-Metz, University of Southern California: dmetz@usc.edu
