# Supplementary material for: Developing and Evaluating Digital Interventions to Promote Behavior Change in Health and Health Care: Recommendations Resulting From an International Workshop
Source: J Med Internet Res. 2017 Jun 29;19(6):e232. doi: 10.2196/jmir.7126 (PMC5509948; doi:10.2196/jmir.7126)
Supplement: Multimedia Appendix 2 [file jmir_v19i6e232_app2.pdf]

Multimedia appendix 2

Options for testing DBCIs.

**Original research**	
Interviews with users	Talking to users with or without props using a structured or semi-structured interview schedule.
Think-aloud	Asking users to articulate their thoughts as they use an intervention or part of one, or a mock up.
Observation of use	Observing users and recording their behaviour as they interact with the intervention.
'Dog-fooding'	This involves developers using their own products to identify bugs and gain a personal sense of whether it is achieving its goals.
N-of-1 studies[1]	These are formal studies in which data are collected over a period of time at frequent intervals and temporal trends are tested as a function of introduction of changes to the user's environment or the product being tested; they may be randomised.
Sequential A-B testing[1]	This involves establishing a baseline for a key set of variables and then making a change to the product and determining what effect this has.
Concurrent A-B testing[1]	This involves giving different versions of a product to different groups of users and establishing what difference this makes.
Uncontrolled quantitative evaluation	Measurement of key outcome and process parameters in a sample of users, typically accompanied by assessment of associations between user characteristics and those measures.
Factorial or fractionated factorial experiments[1]	Experimental studies in which components of an intervention are varied in an orthogonal manner across different randomly allocated groups of users so that they are not confounded with each other and can be evaluated as though they were in separate experiments as long as there are not higher order interactions between them; interactions can also be evaluated.
Non-randomised comparisons	Comparison between users of the product and a comparison group without allocation to the groups being random, or between products that contain different features or components.
SMART designs[1]	Sequential Multiphase Adaptive Randomised Trials (SMART) are ones in which individuals are randomised to receive different products or a product with different features. Then, according to a decision rule, those who respond well are allocated to a new comparison as are those who respond less well or not at all. This process may continue in order to identify efficient and effective interventions for different subgroups.
Trials of Intervention Principles[1]	The concept here is to use experimental studies to compare features of interventions based on the principles underlying them, allowing variation in implementation of these features so that generalisations are made about the features rather

	than their specific implementation.
Cost-impact evaluations	These studies usually use an experimental study to measure the effect of an intervention relative to a comparator and cost information and assumptions about the benefits arising from the effect observed to arrive at a cost-impact assessment.
Randomised controlled trials[1]	This involves randomly allocating users of an intervention, usually with their consent, to receive different versions of an intervention or an intervention versus one or more comparators, and assessing the differences in one or more outcome measures. RCTs usually involve assessment of process variables as well in order to assess the mechanism of action.
**Evidence synthesis**	
Expert review	This involves gathering views from experts, often with discussion, on a set of ideas or proposals.
Meta-analyses	This involves combining data from multiple studies to estimate an overall effect size with confidence intervals on the assumption that the intervention, context and measures of outcome are sufficiently similar to be able to draw general conclusions about the categories of interventions, populations, settings and behavioural outcomes specified. Effect sizes always need to be expressed in terms of defined comparators.
Meta-regression	This involves attempting to explain heterogeneity in effect sizes found in meta-analyses by examining how far these are associated with components of the intervention (e.g. BCTs), differences in the target population, setting or outcome measure. These analyses need to take into account differences in study design and quality.
Classification and regression trees	These are a form of exploratory data analysis in which predictor variables are chosen one at a time to partition the respondents in terms of their outcomes with the process being applied iteratively to each partition. This in theory has the ability to detect interactions between predictor variables.
Content synthesis	This method identifies the content or other features of interventions that have been found to be effective.

[1]Denotes experimental designs in which a researcher introduces variation and observes the result

From: West R, Michie S (2016) A Guide to Development and Evaluation of Digital Behaviour Change Interventions in Healthcare. London: Silverback Publishing.
